# Paraphrase and translation: the importance of being close

**DOI:** 10.12688/openreseurope.19108.1

**Published:** 2025-02-18

**Authors:** Diana Santos, Anabela Barreiro

**Affiliations:** 1 Department of Literature, Area Studies and European Languages, University of Oslo, Oslo, Norway; 2 Linguateca, Oslo, Norway; 3 Instituto de Engenharia de Sistemas e Computadores Investigação e Desenvolvimento em Lisboa, Lisbon, Portugal

**Keywords:** Paraphrase, Translation, Computational Linguistics, Paraphrase Generation, Paraphrase Detection, Closeness, Formalization, Equivalence

## Abstract

This article explores the concept of paraphrasing within computational linguistics, seeking to enrich its understanding by drawing parallels with translation studies and especially machine translation. It highlights the existence of two distinct yet related tasks: paraphrase generation and paraphrase detection, as well as points out the many (sometimes implicit) contact points in evaluation in both translation and paraphrasing. We claim that the concept of near-synonymy or near- equivalence is a shared concern of both disciplines, and its formalization should be pursued.

## Plain language summary

This article examines paraphrasing—a way of expressing the same meaning using different words or phrasing—and its relationship with computational linguistics and translation studies. It focuses on two key tasks in paraphrasing: generation (creating paraphrases) and detection (identifying them). Both tasks are essential for applications like machine translation, summarization, question answering, and plagiarism detection. The study emphasizes how paraphrasing and translation share the concept of near-synonymy or near-equivalence, where slight differences in word choice preserve the same core meaning. While translation involves converting text between languages while keeping the meaning intact, paraphrasing focuses on rephrasing within the same language for different purposes—like adapting for a specific audience or simplifying complex ideas. Despite their differences, both processes require balancing the original intent with contextual adjustments. The article also highlights how concepts from translation studies can inform paraphrasing research. For example, the Portuguese phrase "Por Outras Palavras" ("In Other Words") has been used as the title for both translation and paraphrasing workshops, underscoring their conceptual overlap. By exploring these connections, the study aims to deepen our understanding of paraphrasing and improve methods for creating and detecting paraphrases in natural language processing.

## 1 Motivation

The identification of paraphrases has emerged as a significant area of study in computational linguistics, being applied across various domains such as machine translation, language generation, summarization, text reuse identification, text simplification, question answering and plagiarism detection. Recognizing different ways of expressing the same or a similar meaning is crucial for enhancing the effectiveness of all these applications. Paraphrase identification is also often considered a component of text entailment detection, the task of determining whether one text logically follows from another. This broad applicability highlights the importance of developing robust methods for detecting and generating paraphrases in natural language processing.

In this article, we examine multiple definitions and operationalizations of paraphrases found in the literature, showing two distinct definitions of paraphrases that have emerged in the field – those based on systematic relationships between linguistic expressions out of context and those based on empirically determined semantic equivalence or closeness at the sentence level.

We deem it essential to emphasize the deep connection between paraphrasing and translation, as both hinge on concepts like near-synonymy or near-equivalence, allowing slight differences in word choice or phrasing that remain true to the original intent. Translation aims to maintain semantic equivalence while converting text from one language (the source language) to another (the target language). This process goes well beyond merely swapping words, as it often demands an awareness of different cultural contexts, histories, and linguistic norms to accurately convey meaning, ensure clarity and preserve the original intent of the author. Paraphrasing, in contrast to translation, remains within the same language but may involve rephrasing text to suit different audiences, styles, contexts, or varieties. While it does not require the cross-linguistic shift that translation does, paraphrasing still demands a careful balance of preserving the original meaning while adjusting the expression.

The connection between translation and paraphrasing can be seen in the title of one of translation studies key texts, "In Other Words" ^1^, where paraphrasing is conceptually linked to translation. And the other way around, the Portuguese phrase "Por Outras Palavras" (literally "In Other Words") has served as the title for two paraphrasing workshops ^2, 3^.

### 1.1 Translation versus paraphrasing

Paraphrasing and translation both imply "closeness of meaning", whether in the same or different languages. When considering the significance of translation versus paraphrasing, one might argue that paraphrasing is less essential, given that translations are often needed to bridge language barriers for audiences who do not understand the original language. Paraphrasing may appear simpler since it operates within the same language. Walter Benjamin ^4^ observed that translation involves transferring a text from one cultural and linguistic framework to another, adding layers of complexity that paraphrasing typically does not face. However, when adapting a paraphrased text for different audiences or cultures, it may require adjustments beyond simple rewording. Much like translation, paraphrasing can demand an awareness of cultural contexts.

In any case, what is undeniable and intriguing is that, as discussed in Section 3 ("Interconnections"), there have been many instances where computational linguistics researchers have drawn from work in either paraphrasing or translation without explicitly emphasizing the theoretical closeness between the two. These overlaps have fostered a natural, albeit often unspoken, interconnection between the two disciplines. Finally, the increasing role of large language models (LLMs) in refining and paraphrasing texts has sparked important questions about authorship, originality, and accountability, and this also calls for a deeper understanding of paraphrasing and the development of robust paraphrase detection methods. The goal would be to ensure that the nuances between paraphrased and original content can be accurately identified, addressing concerns related to ownership and academic integrity.

## 2 Paraphrase in computational linguistics

In computational linguistics, tasks related to paraphrasing generally fall into two categories: paraphrase generation and paraphrase detection. These two tasks often imply distinct underlying definitions of paraphrase. Paraphrase generation has typically emphasized competence, i.e., stressing the linguistic system’s ability to produce paraphrases. In contrast, paraphrase detection is more concerned with performance, looking at how paraphrases are identified or recognized in actual language use. While paraphrase generation begins with a singular meaning and explores various ways to express it, paraphrase detection focuses on determining whether two different sentences convey the same meaning or a close enough/approximate meaning.

Starting by paraphrase-related research in the area of language generation, Smadja and McKeown ^5^ explored a system called Cook, which uses extensive collocation knowledge extracted from a domain-specific corpus to generate diverse sentences from what they refer to as "semantic messages". The system’s ability to merge collocations while managing multiple constraints demonstrates a significant potential for generating paraphrases (pp. 238). A notable aspect of their system is its adherence to what they describe as the "delocality of semantic constraints", a concept attributed to Kukich *et al.* ^6^, referring to the phenomenon where the same semantic information is distributed across different parts of the sentence, sometimes separated by considerable distance. Similarly to Smadja and McKeown, Kukich and colleagues had developed a system designed to help engineers generate documentation for telephone network planning operations. Their system demonstrated the flexibility of paraphrase generation by producing an average of 150 different sentence variations for a single message type.

A key development in paraphrase research came from the article by Barzilay and McKeown ^7^, "Extracting Paraphrases from a Parallel Corpus". They introduced a performance-based view of paraphrasing, which has since become the dominant approach. This shift in perspective emphasized the practical application of paraphrase detection by using large datasets, such as parallel corpora – already used in translation – to extract paraphrased pairs and better understand their variations in real-world contexts. They emphasized that existing resources predominantly focused on lexical paraphrases, i.e., alternative expressions at the word level, noting the absence of phrasal or syntactically-based paraphrases. In their view, this limited the scope of how paraphrases are understood and used in computational tasks. According to these authors, previous approaches used manual collection of paraphrases for generation, something which is time consuming and application-specific; or a) used lexical resources such as WordNet (synonymy and/or other relations) to compute paraphrases; b) employed morphological and syntactical processors applied to term lists, what they call "term paraphrases" ^1^; or c) used (linguistic) explanations of the meaning of regular polysemous adjectives ^8^. In all these three latter cases, they claim that the process may result in unnatural/unattested examples, and this is why they propose to identify actually used paraphrases. However, Barzilay and McKeown ^7^ faced a significant challenge in evaluating the paraphrases generated by their method, because they had to rely on human judgment. They defined a paraphrase as demonstrating "approximate conceptual equivalence" but encountered difficulty in determining whether the evaluation should involve context or not. In some cases, only by considering the context could one judge whether two phrases were indeed paraphrases. But, including the context meant that the paraphrases would become dependent on specific situations, reducing their generalizability. Context-dependent paraphrases could not be used across different settings, making them less valuable as general linguistic resources. On the other hand, excluding context in evaluations risked missing nuances, as paraphrases might only work as substitutes in certain contexts. The challenge then was whether to include larger text snippets to capture context more effectively, an approach which would result in longer paraphrase pairs – not explored in their paper. One can clearly see that a trade-off exists between generalizability and accuracy in determining paraphrastic relationships. We believe their paper was a cornerstone for the paraphrase detection task, which turned into a larger endeavour in the years to follow.

Dolan and Brockett ^9^ developed the Microsoft Research Paraphrase Corpus (MRPC) using a significantly large data set, employing two key techniques on clustered news articles. The first involved finding pairs of sentences with a relatively small Levenshtein distance ^10^, a measure of the difference between two sequences by counting the minimal number of single-character edits required to transform one sentence into another. The second technique focused on identifying the initial sentences of articles with considerable lexical overlap and similar sentence lengths, as these similarities increased the likelihood that the sentences were paraphrases of one another. The MRPC corpus ^2^, containing 5,800 sentence pairs, was released in 2005 with human judgments indicating whether each pair was a paraphrase or not. Since its creation, the MRPC has become an invaluable resource for the development and evaluation of paraphrase detection models. As detailed by Finch *et al.* ^12^, these human judgments assessed whether the sentences were close enough in meaning to be considered paraphrases. Two annotators independently labeled the data, and a third annotator resolved disagreements. The inter-annotator agreement was 83%, underscoring both the complexity of the task and the ambiguity in labeling, with about 67% of sentence pairs classified as paraphrases. Once again, we have genuine sentence pairs conveying the same core information, but human verification is required to ensure this alignment. Given the wealth of this resource, Finch *et al.* ^12^ explored the effectiveness of evaluation metrics typically used in machine translation to assess "semantic equivalence classification" at the sentence level, considering paraphrases as semantic equivalence data. They also used data from the PASCAL challenge ^13^, which focused on textual entailment. In this context, sentences were classified as entailing (or not) the other sentence in the pair. Mutual/double entailment between sentences indicated a paraphrase. Like the MRPC, PASCAL judgments were human-validated.

Plagiarism detection corpora have also been created, as noted by Potthast *et al.* ^14^, who differentiated between real, simulated, and artificial forms of plagiarism. Although plagiarism and text reuse have distinct characteristics, they can still be seen as a form of paraphrase, as suggested by Madnani *et al.* ^15^, who use machine translation evaluation metrics to assess the precision of paraphrase detection. In another study, Gupta *et al.* ^16^ examine techniques for detecting crosslingual plagiarism, particularly when machine translation is used to plagiarize text written in another language. This form of plagiarism introduces unique challenges, as content is disguised through translation.

Over time, several paraphrase datasets have been compiled, many of which were based on translation, following the pioneering ideas of Barzilay and McKeown ^7^, but on a much larger scale. For example, Wieting and Gimpel ^17^ released a dataset containing 50 million paraphrased sentences.

Recently, this understanding has led to the annotation of paraphrase pairs at the phrase level. For example, Wang *et al.* ^18^ introduced ParaTag, a dataset specifically designed to capture fine-grained paraphrasing at the constituent phrase level. Interestingly, their study revealed that in almost 90% of instances labeled as paraphrases in the MRPC dataset, at least one phrase was missing from the paraphrased version. This indicates that while the general meaning remained, parts of the original sentence were frequently left out. Researchers have thus returned to emphasizing phrases after previously shifting from individual words and phrases to entire sentences.

Another important methodological breakthrough was brought in 2005 by Callison-Burch and associates. Bannard and Callison-Burch ^19^, Callison-Burch ^20^ used parallel corpora to find paraphrases, asserting that this resource is far more prevalent than multiple translations as proposed by Barzilay and McKeown, on the one hand, and, on the other hand, simpler to process than monolingual corpora, which require parsing and clustering, as used by Lin and Pantel ^21^. Their approach offers a statistical measure to evaluate the quality of paraphrase candidates. Later, Callison-Burch and colleagues demonstrated the benefits of using paraphrasing to enhance machine translation performance ^22^. In 2013, they released a large paraphrase database in English and Spanish ^23^, annotated with several contextual features and evaluation information.

After Callison-Burch doctoral studies, Barreiro’s research ^24^ introduced the notion of bilingual/multilingual paraphrases, arising from aligning texts across one language pair (bilingual) or multiple language pairs (multilingual). She proposed the concept of a resource similar to a dictionary but designed for paraphrases at the multiword and phrasal levels, naming it "paraphrasary" ("parafrasário", in Portuguese). Using a revised version of Callison-Burch’s Linear-B tool, called CLUE-Aligner ^25^, her approach identifies manually a set of paraphrastic units within parallel sentences, enabling analysis of equivalences not only across languages but also within Portuguese language varieties ^26, 27^. The methodology, further refined, detects non-contiguous paraphrastic units and aligns them for both paraphrastic and translation unit pairs ^28^.

Paraphrase evaluation faces two primary challenges: one concerning the assessment of semantic importance and the other regarding the influence or dependence of context. As Dras ^29^ aptly put it, paraphrases involve "a pair of text units considered interchangeable". But how to define what is interchangeable?

First, not all parts of a sentence carry the same weight when it comes to recognizing semantic equivalence or non-equivalence, as Madnani *et al.* ^15^ pointed out. Rather than viewing paraphrastic relationships as strictly binary, they suggested using a continuous scale between 0 and 1 to reflect varying degrees of similarity.

Another challenge is the issue of context. Assessing phrases or words outside their original context requires careful consideration of suitable independent contexts. The idea of simply relying on the original context, as done by Barzilay and McKeown, or Mota *et al.*, has faced criticism, notably from Callison-Burch in his PhD thesis. He argued that this method is too lenient, as it may permit phrases to be judged as paraphrases without adequately testing their versatility across different contexts.

The evaluation of paraphrases ranges from wholly subjective methods, where individuals rate or rank paraphrases based on their perceived closeness, to more "objective" approaches that involve quantifying the frequency of words identified as paraphrases across various texts and languages. Other techniques include analyzing the similarity of n-grams or lexical units between sentences, or using word embeddings. However, a central question persists: is there a universally accepted definition of what constitutes a paraphrase? Despite progress in these evaluation methods, reaching a consensus on this issue continues to be a challenge.

## 3 Interconnections between paraphrasing and translation

In this section, we pinpoint and elucidate the diverse connections between paraphrasing and translation within computational linguistics, classifying them in five cases: (1) using translation to obtain paraphrases; (2) using paraphrases to improve translation; (3) using paraphrases to evaluate machine translation; (4) using machine translation evaluation metrics to evaluate paraphrases; and (5) combining/mixing translation and paraphrasing in the same task.

Case 1 represents one of the most straightforward connections between paraphrasing and translation: variations that emerge from different (human) translations of the same source text. These translations naturally serve as paraphrastic candidates, when they occur at the sentence level, as shown by Barzilay and McKeown’s work, or through retranslation of the same phrase or word across bilingual corpora, as Callison-Burch has proposed. Wieting and Gimpel ^17^ leveraged Czech-English machine translation using an English-Czech bilingual corpus to increase the volume of English paraphrases available.

Case 2 focuses on using paraphrases to improve (machine) translation quality by offering multiple ways to express the same meaning. As early as 1990, Santos [ 30, pp. 330] suggested using paraphrasing as a final step in the machine translation process to enhance output quality. The translation process was divided into two phases: "structural transfer", which performed the primary translation, and "style transfer", which involved selecting paraphrases in the target language ^3^.

Callison-Burch was the first to actually demonstrate the use of paraphrases to improve coverage in statistical machine translation (SMT). When direct translations are unavailable, paraphrases can fill the gap using existing translations from bilingual corpora, potentially across multiple languages. His approach enhanced statistical systems by expanding the number of translation options.

Barreiro ^24^ studied the efficiency of using short paraphrases of support verb constructions to enhance machine translation. She developed eSPERTo, a tool for paraphrasing and translation tasks, originally known as SPIDER ^31^, incorporating lexicon-grammar resources within a human-in-the-loop framework ^32^. Then, Barreiro and colleagues introduced a method for enhancing machine translation by using monolingual parallel corpora from two varieties of Portuguese, as presented in Barreiro and Mota ^26^.

Case 3 provides another crucial link between paraphrasing and translation research, namely the use of paraphrases to evaluate machine translation. Notably, BLEU ^34^, a well-known machine translation evaluation metric, uses reference translations treated as paraphrases to assess machine-generated translations, measuring their closeness to human translations. As Madnani *et al.* [ 15, pp. 182] note:

the task of an MT metric is essentially one of identifying whether the translation produced by a system is a paraphrase of the reference translation.

Unlike typical paraphrase evaluations, which often consider individual paraphrases, BLEU evaluates translations comparatively. While guidelines for diversity in reference paraphrases are lacking, researchers have explored various facets of evaluating paraphrases in their respective studies. In 2023, Kanayama ^35^ introduced a novel approach to evaluating Japanese machine translation output by proposing the use of paraphrasing rules instead of traditional n-gram based evaluation metrics.

Case 3 transitions into Case 4, where machine translation evaluation metrics are repurposed to assess the quality of paraphrases. This shift leverages the metrics typically used for translation evaluation to gauge the similarity and effectiveness of paraphrased expressions, helping measure the degree of semantic alignment between original and paraphrased texts. Quirk *et al.* ^36^ introduced the concept of "monolingual machine translation", while Finch *et al.* ^12^ adapted BLEU for paraphrase evaluation. Similarly, Wan *et al.* ^37^ combined BLEU’s features with dependency analysis to improve paraphrase assessment. Building on these efforts, Madnani *et al.* ^15^ broadened this scope by applying eight different machine translation evaluation metrics to evaluate paraphrase identification. These works highlight the growing inter-section between paraphrase assessment and machine translation evaluation, producing another implicit argument for the closeness of both tasks.

Finally, case 5 includes broader applications that integrate both translation and paraphrasing without any hierarchical preference. An example is the multilingual plagiarism detection system discussed by Gupta *et al.* ^16^, which employs both translation and paraphrasing techniques.

## 4 Closeness of meaning

Both translation and paraphrasing are intrinsically linked to the idea of "closeness" or "sameness" of meaning. Over time, several researchers in paraphrase have invoked sameness. Kukich *et al.* ^6^ discussed the concept of paraphrases sharing "the same semantic information". Barzilay and McKeown ^7^ noted that "paraphrases are different ways of expressing the same information". Finch *et al.* ^12^ referred to paraphrasing as "Sentence-level Semantic Equivalence". Wieting *et al.* ^38^ described a paraphrase table as containing pairs of text snippets "that have the same meaning". And Globo *et al.* ^39^ stated that paraphrasing is rewriting "without altering the meaning of the original content".

Others emphasized closeness of meanings: Barzilay and McKeown ^7^ described paraphrases as having "approximate conceptual equivalence". Finch *et al.* ^12^ discussed whether two sentences are "close enough in meaning".

Qiu *et al.* ^40^ argued that paraphrase recognition (PR) operates on the concept of "relaxed meaning", which allows sentence pairs with minor variations to still qualify as paraphrases. They proposed that PR can be understood as an extension of synonym detection, but at the sentence level, and acknowledged that absolute synonymy is rare in natural language, as nuances of meaning are frequently added or removed in the paraphrasing process. They claimed:

[...] for many people the notion of paraphrases also covers cases in which minor or irrelevant information is added or omitted in candidate sentences, [...]. Such extraneous content should not be a barrier to PR if the main concepts are shared by the sentences.

So we conclude that the challenge lies in identifying which elements constitute the "core concepts" and distinguishing them from details that can be safely omitted or added without altering the fundamental meaning. We thus turn to research that seeks to formalize the concept of "closeness of meaning".

Edmonds and Hirst [ 41, pp. 111] introduced the idea of "near-synonymy" precisely when addressing translation. They argued that the closest word in the target language is often a near-synonym rather than an exact synonym.

Near-synonymy rather than synonymy is the norm in lexical transfer in translation: the word in the target language that is closest to that in the source text might be a near-synonym rather than an exact synonym.

Already in 1967, Catford ^42^ had observed that translations often do not convey the exact same message, but instead use different linguistic resources to achieve a comparable outcome.

Kay ^43^, cited in Edmonds and Hirst ^41^, introduced the concept of "elementary meanings", the core semantic units of a sentence, stripped of peripheral elements such as connotations or idiomatic quirks: "discrete objective denotations uncolored by [...] peripheral aspects such as connotations, implications, or quirks of idiomatic usage".

Edmonds and Hirst ^41^ extended Kay’s concept by proposing a model for encoding near-synonymy, presented as a key characteristic of natural language. Their model consists of two components: (1) a language-neutral ontology of coarse-grained representations, essentially clusters of near-synonyms that are context-independent; and (2) a set of differences between these near-synonyms that capture relations to context, fuzziness, and degrees of necessity. These differences, which they termed a "subconceptual level", highlight subtle variations between words that go beyond core meaning. In their model, these differences are expressed through concepts, attitudes, and stylistic dimensions, which can vary across languages. This representation of near-synonymy accommodates a wide range of linguistic distinctions without limiting the kinds of nuances that can be conveyed. Their graphically illustrated examples (e.g., distinguishing between the English nouns *blunder* and *error*, and the English verbs *order* and *enjoin*) show that encoding such distinctions requires a substantial amount of information, which they acquired from existing thesauri.

From a different perspective, but along similar lines, Wierzbicka’s Natural Semantic Metalanguage (NSM) approach, as discussed in works like Goddard and Wierzbicka ^44^, Wierzbicka ^45^, and Wierzbicka ^46^, sought to express complex meanings by breaking them down into configurations of semantic elements using a limited set of basic, universally translatable words known as semantic primes. Interestingly, the defense of this approach also draws on the concept of paraphrasing:

The NSM analysis of meaning is based on "reductive" paraphrase, in the sense that complex meanings are "reduced", in a systematic way, to simple or simpler ones. It attempts to say "the same thing" in a paraphrase composed of maximally simple, intelligible, and cross-translatable words (semantic primes), thereby laying bare the semantic content compressed in the original expressions.

Finally, another approach to handling complex meanings, from very distant quarters, is the method used for answering definition questions through informational "nuggets", as introduced by the QA track of TREC ^47^. In this framework, the evaluation of an automated response to a complex question is based on how effectively it captures key pieces of information, or "nuggets", that are deemed essential to a complete and accurate answer (and which were previously listed by the organizers).

We thus note that scholars like Edmonds and Hirst, Wierzbicka and Voorhees have developed (different) fine-grained meaning representations, which should enable a more precise understanding of both translation and paraphrasing. In all of them, meaning proximity is crucial, but discrete, and we believe this is the way to proceed.

## Ethics and consent

We confirm that this study did not involve interviews, workshops, or surveys with individuals, ensuring adherence to ethical standards for research involving human subjects.

## Data Availability

No data or software were developed in this study.
