# Supplementary material for: Paraphrase and translation: the importance of being close
Source: Open Res Eur. 2025 Feb 18;5:52. [Version 1] doi: 10.12688/openreseurope.19108.1 (PMC11904397; doi:10.12688/openreseurope.19108.1)
Supplement: Peer review history

# Reviewer report: Reviewer response for version 1

**DOI:** 10.21956/openreseurope.20679.r51650

**Published:** 2025-03-12

**Authors:** Eman Abdel Reheem Amin

**Affiliations:** 1 Majmaah University, Al Majma'ah, Saudi Arabia

**Summary of the Article:**

 The article explores the intricate relationship between paraphrasing and translation, emphasizing the necessity of maintaining semantic and stylistic proximity to the original text. It presents a well-researched discussion on how paraphrasing can either support or challenge translation fidelity, particularly in contexts where linguistic and cultural nuances play a critical role.

 The author systematically examines existing literature, theoretical frameworks, and real-world examples, offering a thorough and insightful analysis of the topic. this article is a well-structured, well-researched, and clearly articulated contribution to the study of paraphrasing and translation.

 The discussion is thorough, the arguments are well-supported, and the conclusions are appropriately drawn. No major concerns were found, and the paper is well-positioned to contribute valuable insights to the field.

 I recommend the article for indexing in bibliographic databases after standard editorial checks.

Does the review offer a comprehensive analysis of the research literature?

Yes

Are all statements factually correct and adequately supported by citations?

Yes

Is the review written in clear language?

Yes

Are the conclusions drawn appropriate in the context of the current literature, books and book chapters?

Yes

Reviewer Expertise:

Applied lingustics

I confirm that I have read this submission and believe that I have an appropriate level of expertise to confirm that it is of an acceptable scientific standard.

# Reviewer report: Reviewer response for version 1

**DOI:** 10.21956/openreseurope.20679.r51654

**Published:** 2025-03-11

**Authors:** Stella Markantonatou

**Affiliations:** 1 Athena Research Center, Athena, Greece

The paper makes an interesting review of the literature on paraphrasing in computational linguistics with two main goals, one explicitly stated and one served implicitly. Explicitly stated is the goal of highlighting  the conceptual similarities of paraphrasing and translation, as they both start from a “given” text and try to develop texts that simultaneously differ in form from the “given” one and convey the same meaning with it. Of course, translation works across languages while paraphrasing within the same language as the “given” text, which may simplify the task to some extend. The implicit goal is to identify the features of good paraphrases and develop a theory for them; the authors seem to consider theories relying on semantic primitives as a proposal to this end.  

 The authors go through important points in the history of paraphrasing in computational linguistics and explain in sufficient detail how the field evolved following the developments in NLP and came close to research on translation and eventually shared with it materials and evaluation techniques. In particular, NLP techniques based on parallel corpora allowed for searching for the  naturally occurring  different textual forms in a corpus of language A that were mapped on to the same translation in a language B; these texts were evaluated by humans as to whether they constituted paraphrases of each other. This idea was refined with various techniques and evaluation measurements but the main problem remained: could the texts evaluated as paraphrases substitute one another in different contexts? Which properties of the texts would determine substitutability? In simpler and somehow more vague terms, which properties of the texts are responsible for good paraphrases? This is what we have called the implicit goal of the article.

 Paraphrasing relies on the hard-to-define notion of “sameness” or “equivalence” of meaning carried by texts different in form. An important finding is that the parts of  paraphrases may not stand in 1:1 relation among them, rather some parts are more important while others may be omitted.  A theory of “sameness” should be able to explain such observations. The authors believe that theories  based on semantic primitives allowing for a direct description of meaning, are best candidates for this purpose. Theories of semantic primitives, though, have never managed to achieve wide acceptance because they failed to convince that the defined primitives were enough or the right ones. This is a very old problem, that has motivated various approaches of indirect description of word meaning, e.g.,  WordNet uses Synsets (where meaning is described by lists of synonyms) while in state-of-the-art NLP, embeddings describe word/phrase meaning by the contexts in which a word/phrase occurs.

 We would like to make two observations. First, the article makes an unnecessary, in our view, reference to the Chomskyan distinction between competence and performance. These notions apply to human linguistic abilities and not to computational systems, unless the authors imply that the systems they examine somehow simulate human cognition. The distinction between paraphrase generation and paraphrase discovery, also made by the authors, is enough and suitable for the systems that the paper examines. And second, the paper does not put its topic under the perspective of LLMs.  Are paraphrases interesting in a world where LLMs dominate NLP or are they a necessity of the technology before LLMs?

 In the overall, this is an informative and clearly written article, that conveys well-organised information even if its readers do not agree with the generalisations and the preferences of the authors.

Does the review offer a comprehensive analysis of the research literature?

Yes

Are all statements factually correct and adequately supported by citations?

Yes

Is the review written in clear language?

Yes

Are the conclusions drawn appropriate in the context of the current literature, books and book chapters?

Yes

Reviewer Expertise:

NLP, ontologies

I confirm that I have read this submission and believe that I have an appropriate level of expertise to confirm that it is of an acceptable scientific standard.
